# Student-directed retrieval practice is a predictor of medical licensing examination performance

**DOI:** 10.1007/s40037-015-0220-x

**Published:** 2015-10-23

**Authors:** Francis Deng, Jeffrey A. Gluckstein, Douglas P. Larsen

**Affiliations:** 1 Washington University School of Medicine, Campus Box 8077, 660 S. Euclid Ave, 63110-1093 Saint Louis, MO USA; 2 Department of Neurology, Washington University School of Medicine, Saint Louis,, MO USA

**Keywords:** Spaced repetition, Licensing examinations, Test-enhanced learning, Self-directed learning

## Abstract

**Introduction:**

A large body of evidence indicates that retrieval practice (test-enhanced learning) and spaced repetition increase long-term information retention. Implementation of these strategies in medical curricula is unfortunately limited. However, students may choose to apply them autonomously when preparing for high-stakes, cumulative assessments, such as the United States Medical Licensing Examination Step 1. We examined the prevalence of specific self-directed methods of testing, with or without spaced repetition, among preclinical students and assessed the relationship between these methods and licensing examination performance.

**Methods:**

Seventy-two medical students at one institution completed a survey concerning their use of user-generated (Anki) or commercially-available (Firecracker) flashcards intended for spaced repetition and of boards-style multiple-choice questions (MCQs). Other information collected included Step 1 score, past academic performance (Medical College Admission Test [MCAT] score, preclinical grades), and psychological factors that may have affected exam preparation or performance (feelings of depression, burnout, and test anxiety).

**Results:**

All students reported using practice MCQs (mean 3870, SD 1472). Anki and Firecracker users comprised 31 and 49 % of respondents, respectively. In a multivariate regression model, significant independent predictors of Step 1 score included MCQs completed (unstandardized beta coefficient [B] = 2.2 × 10^− 3^, *p* < 0.001), unique Anki flashcards seen (B = 5.9 × 10^− 4^, *p* = 0.024), second-year honours (B = 1.198, *p* = 0.002), and MCAT score (B = 1.078, *p* = 0.003). Test anxiety was a significant negative predictor (B= − 1.986, *p* < 0.001). Unique Firecracker flashcards seen did not predict Step 1 score. Each additional 445 boards-style practice questions or 1700 unique Anki flashcards was associated with an additional point on Step 1 when controlling for other academic and psychological factors.

**Conclusions:**

Medical students engage extensively in self-initiated retrieval practice, often with spaced repetition. These practices are associated with superior performance on a medical licensing examination and should be considered for formal support by educators.

## Introduction

In traditional education paradigms, students learn by studying, whether by attending lectures, reading, or taking notes. Students are then tested for the purpose of measuring knowledge acquisition. Substantial evidence has shown that the act of recalling information from memory through questions or testing causes the memory of that information to be strengthened, a principle known as retrieval practice [1]. Repeated testing produces larger positive effects on delayed recall compared with restudying for an equivalent duration [2]. Even a single test can promote retention [3].

Medical education researchers have taken interest in retrieval practice, also known as test-enhanced learning [4]. For instance, repeated testing of residents over a month resulted in greater retention of information from a didactic conference than spaced studying, as assessed more than 6 months after the initial teaching [5]. In another study of first-year medical students, repeated testing was superior to self-generating explanations for six-month retention [6]. Other groups have harnessed technology to provide spaced retrieval practice for medical students via weekly emails [7], electronic flashcards [8], and quizzes within an online course management system [9].

Despite these promising experiments, tests in formal medical curricula are largely implemented as one-time assessments or motivators for studying, rather than as mnemonic enhancers. Testing need not be teacher-directed, but reports of student-directed testing have been scant as well. An older survey found that second-year students infrequently used practice test questions and flashcards to prepare for problem-based learning examinations and these study aids did not correlate with performance [10]. In our experience, however, we observed that students are more likely to choose these study methods for high-stakes examinations that depend on retaining a more comprehensive body of knowledge.

The culminating opportunity to demonstrate retention for US preclinical medical students is Step 1 of the United States Medical Licensing Examination (USMLE), a cumulative assessment of basic medical sciences. Performance on this eight-hour, multiple choice exam is the most common factor used by residency programme directors to select applicants for interviews [11]. Understandably, there is great interest in clarifying effective study strategies. While general self-testing behaviours as measured on the Learning and Study Strategies Inventory was not a significant predictor of Step 1 performance [12], completion of more items from commercial question banks has been associated with higher Step 1 scores [13]. Similarly, students who completed more forms of the National Board of Medical Examiners Comprehensive Basic Science Self-Assessment (NBME CBSSA) subsequently performed better on Step 1, an effect that the authors attributed to spaced practice [14].

Anecdotally, we perceived that many medical students prepared for Step 1 harnessing retrieval practice not only using boards-style question banks and simulated exams, but also using spaced repetition systems. Question banks present students with a computer-based simulation of the USMLE testing system. The questions typically involve brief clinical vignettes that require multiple steps of retrieval. For instance, a question stem might provide a set of signs and symptoms and then ask for the molecular mechanism of the first-line treatment for the condition described. In contrast, typical spaced repetition systems that are currently available utilize brief, one-step flashcards that prompt active recollection of short answers. After viewing the correct answer for a flashcard, users rate the difficulty of recall. Reviews are scheduled after each viewing to promote long-term retention, but the intervals between viewings are based on difficulty. Anecdotally, the most prevalent spaced repetition systems at our institution were Anki and Firecracker. Anki is a free, open source, multimedia flashcard programme in which users create and share their own flashcards [15]. Firecracker, by comparison, is a commercial web platform that provides pre-made questions geared towards USMLE preparation for a subscription fee [16]. There has been little documentation of the use of these systems in the education literature, as their adoption has spread only in recent years.

The aim of this exploratory study was to measure the use of independent self-testing methods among preclinical students and determine their relationship with USMLE Step 1 performance and measures of student stress. Our primary hypothesis was that the use of practice questions and spaced repetition flashcards is associated with superior Step 1 performance, independent of other academic factors and measures of mental well-being.

## Methods

Students at Washington University School of Medicine in St. Louis who completed the two-year preclinical curriculum in 2014 and had taken Step 1 were eligible for the study. Students were invited to take an anonymous, non-incentivized, web-based survey in August 2014. The student-developed survey was administered as part of routine internal monitoring of student study strategies. Because the present study involved retrospective analysis of de-identified data collected for another purpose, the Washington University Human Research Protection Office determined that this analysis did not require further institutional review board approval.

In the survey, students were asked about their study practices prior to taking Step 1 with respect to the following: ever using Anki, Firecracker, Osmosis, Memorang, or other spaced repetition systems; duration of use of Anki and number of unique Anki flashcards seen; duration of use of Firecracker and number of unique Firecracker flashcards seen; and duration of use of boards-style multiple-choice questions and number of these practice questions completed, including repetitions of questions. To guide estimation of flashcards or questions completed, students were provided with the total number of items available in each of the major commercial products, including Firecracker, NBME CBSSAs, UWorld Step 1 Qbank with Self-Assessment Exams, Kaplan Step 1 Qbank, and USMLE-Rx Step 1 Qmax. Anki users could determine a count of the flashcards in their collections and usage over time via the application’s statistics tool. To prompt recall of duration of use, students were reminded that the second year curriculum spanned 43 weeks. Performance data collected included the three-digit score on USMLE Step 1, numerical composite score on the Medical College Admission Test (MCAT) out of 45, and number of honours grades out of 17 second-year courses. To assess the representativeness of our sample, Cohen’s standardized difference *d* was calculated for exam scores reported for survey respondents compared with institutional data on the entire class. By conventional criteria, Cohen’s *d* of 0.2–0.5 is small, 0.5–0.8 is moderate, and ≥ 0.80 is large.

The survey included brief measures of depressive symptoms, burnout symptoms, and test-related anxiety. On a frequency scale from 0 (never) to 6 (every day), students indicated how often during the period when they were seriously preparing for Step 1 they felt depressed mood (‘down, depressed, or hopeless’), anhedonia (‘having little interest or pleasure in doing things’), emotional exhaustion (‘burned out from my work’), or depersonalization (‘more callous toward people’), using phrasing adapted from ultra-short versions of the Patient Health Questionnaire and the Maslach Burnout Inventory [17]. On a Likert scale from 1 (strongly disagree) to 5 (strongly agree), students indicated their agreement with two statements about worry when taking tests in general (‘I seem to defeat myself while working on important tests’ and ‘During examinations, I get so nervous that I forget facts I really know’). These statements were derived from a short form of the Test Anxiety Inventory worry subscale [18]. The worry subscale was chosen because it has been correlated with performance on NBME Part I, the precursor to USMLE Step 1 [19]. Cronbach’s alpha reliability coefficients were calculated to determine internal consistency within these pairs of questions.

Statistics were analyzed using XLSTAT software. The analyses included descriptive statistics, a correlation matrix, and multiple linear regression. Correlations were computed as Pearson’s r coefficients. Differences in use of Anki compared with Firecracker were analyzed by Mann-Whitney U tests. Partially completed surveys were omitted altogether in the multivariate regression (listwise deletion) but were included to the extent possible in the univariate or bivariate analyses (pairwise deletion). There were 7 such responses, which left blank either the number of questions or flashcards completed. These omitted responses did not differ significantly from the included responses on any of the remaining variables. For the purposes of descriptive statistics, students were counted as a user of a specific programme if they reported a positive number for either weeks used or flashcards seen.

In the regression model, the dependent variable was Step 1 score. The independent variables were MCAT score, number of second-year honours grades, number of boards-style practice questions completed, number of Anki flashcards seen, number of Firecracker flashcards seen, summed score on the depressive symptom rating items, summed score on the burnout rating items, and summed score on the test anxiety rating items. The numbers of weeks spent using Anki, Firecracker, and boards-style multiple choice questions were excluded from the model due to significant collinearity with the numbers of questions or flashcards completed. An α level of 0.05 was used to determine significance.

## Results

Out of 111 eligible students, 72 completed the survey (65 % response rate). Cronbach’s alpha for the pairs of depressive, burnout, and test anxiety items were 0.86, 0.67, and 0.62, respectively, which were considered acceptable for our exploratory purposes. Means and standard deviations for exam data, academic data, use of testing-based study methods, and measures of well-being and test anxiety are reported in Table 1. Average exam scores reported by respondents were higher than institutional data on the entire class, but differences were small for the USMLE Step 1 (Cohen’s *d* = 0.46) and trivial for the MCAT (Cohen’s *d* = 0.17).

**Tab. 1:** Usage of Anki (*n* = 22) and Firecracker (*n* = 35) limited to the students who used each spaced repetition programme

	Anki^a^	Anki-Wk	Firecracker^a^	Firecracker-Wk
**Mean**	5086	24.5	4588	21.2
**SD**	5719	22.5	3946	16.4

^a^Number of unique flashcards seen.

*Wk* number of weeks spent using a programme.

Every student who responded to the survey used boards-style practice questions in their preparation for Step 1. Sixty-nine percent of students reported having used a spaced repetition programme: Anki users comprised 31 % of respondents, Firecracker users comprised 49 %, and 10 % tried both. Among only those students who used Anki or Firecracker, the typical quantity of unique flashcards seen and duration of use were not significantly different between the two groups of users (Table 2, *p* > 0.05).

**Tab. 2:** Item means, standard deviations, and inter-item Pearson correlation coefficients (*n* = 72)

	Step 1	MCAT	Honours	MCQ^a^	Anki^a^	FC^a^	MCQ-Wk	Anki-Wk	FC-Wk	Burnout	Depr	Mean	SD
**Step 1**												253.6	10.8
**MCAT**	**0.558**											37.4	2.9
**Honours**	**0.603**	**0.320**										14.8	3.2
**MCQ** ^a^	**0.253**	− 0.047	0.073									3870	1471
**Anki** ^a^	**0.264**	0.167	0.163	− 0.093								1346	3661
**FC** ^a^	0.081	0.074	0.038	0.179	− 0.227							2091	3503
**MCQ-Wk**	0.207	0.064	0.095	**0.316**	0.144	**0.272**						19.9	10.7
**Anki-Wk**	**0.279**	0.166	0.176	− 0.103	**0.859**	− 0.200	**0.264**					7.5	16.7
**FC-Wk**	− 0.010	− 0.103	− 0.029	**0.279**	**− 0.245**	**0.782**	0.155	**− 0.260**				10.3	15.6
**Burnout**	− 0.165	− 0.052	− 0.174	0.046	− 0.197	− 0.032	**− 0.233**	**− 0.238**	0.175			5.6	3.3
**Depr**	**− 0.242**	− 0.007	**− 0.474**	− 0.025	− 0.224	− 0.160	**− 0.314**	**− 0.240**	− 0.072	**0.694**		3.6	3.4
**TAnx**	**− 0.557**	**− 0.435**	**− 0.388**	0.085	− 0.010	− 0.072	− 0.061	− 0.108	− 0.019	0.186	0.157	4.0	1.8

*MCQ* boards-style multiple choice questions.

^a^Number of flashcards or questions completed.

*FC* Firecracker, *Wk* number of weeks spent using a programme or question resource, *Depr* depressive symptoms, *TAnx* test-related anxiety.

Values in bold differ from 0 with a significance of *p* < 0.05.

In bivariate correlations with Step 1 score (Table 1), the variables that were significantly related included MCAT score, number of honours grades, number of boards-style multiple choice questions, and number of Anki questions (*p* < 0.05). Test anxiety negatively correlated not only with performance on Step 1, but also in preclinical courses and MCAT (*p* < 0.001). The correlation between Firecracker use and Step 1 performance did not reach statistical significance (*p* = 0.51).

A multiple linear regression model using eight independent variables accounted for 67.2 % of the variance in Step 1 scores (Table 3). There were five statistically significant predictors, which were number of boards-style practice questions completed (unstandardized beta coefficient [B] = 2.2 × 10^− 3^, *p* < 0.001), number of unique Anki flashcards seen (B = 5.9 × 10^− 4^, *p* = 0.024), second-year honours (B = 1.198, *p* = 0.002), MCAT score (B = 1.078, *p* = 0.003), and test anxiety (B = − 1.986, *p* < 0.001). Completing an additional 445 boards-style practice questions or 1700 unique Anki flashcards was associated with one additional point on Step 1 when controlling for the included prematriculation, academic, and psychological factors.

**Tab. 3:** Coefficients for the multivariate linear regression model on Step 1 score

	B coefficient	95 % CI	Standardized β	95 % CI	*p*-value
Honours	**1.198**	(0.468, 1.93)	**0.347**	(0.136, 0.559)	0.002
MCAT	**1.078**	(0.374, 1.78)	**0.280**	(0.0973, 0.463)	0.003
MCQ^a^	**2.25 × 10** ^− 3^	(1.05 × 10^− 3^, 3.44 × 10^− 3^)	**0.298**	(0.139, 0.457)	< 0.001
Anki^a^	**5.89 × 10** ^− 3^	(8.00 × 10^− 5^, 1.10 × 10^− 3^)	**0.195**	(0.0265, 0.3630)	0.024
FC^a^	7.95 × 10^− 5^	(− 4.46 × 10^− 4^, 6.05 × 10^− 4^)	0.0251	(− 0.141, 0.191)	0.763
Burnout	− 0.209	(− 0.986, 0.568)	− 0.0620	(0.292, 0.168)	0.592
Depression	0.233	(− 0.667, 1.13)	0.0693	(− 0.198, 0.337)	0.607
Test anxiety	**− 1.99**	(− 3.12, − 0.857)	**− 0.326**	(− 0.512, − 0.141)	< 0.001

Bolded values differ from 0 with a significance < 0.05, Goodness of fit: R^2^ = 0.672, adjusted R^2^ = 0.625. Analysis of variance of model: df = 8, F = 14.340, *p* < 0.0001.

^a^Number of flashcards or questions completed.

## Discussion

In a cohort of preclinical students at an elite US medical school, retrieval practice was a prevalent study strategy. All survey respondents used boards-style multiple choice questions from commercial question banks and self-assessment exams. The high number of practice questions completed (mean 3870) suggested that most students used multiple question resources or repeated questions. The majority of students also used a spaced repetition system such as Anki or Firecracker with thousands of flashcards. For each of these self-testing methods, the average user started more than 20 weeks prior to their Step 1 exam date, well before the month-long protected study period that follows the end of preclinical coursework. These results demonstrate the popularity of long-term retrieval practice that supplements the formal curriculum.

Though students dedicate considerable energy to studying outside of medical school coursework, there have been few proven predictors of performance on preclinical licensing exams outside of pre-matriculation factors such as the MCAT exam [20, 21], curricular indicators such as second-year grades [12, 22], and measures of test anxiety [19, 23]. For instance, participation in exam coaching courses was not independently associated with higher Step 1 scores [22, 24, 25], nor was use of commercial study guides, after controlling for pre-matriculation factors [24]. In this study, we identified the self-directed use of boards-style questions and Anki, but not Firecracker, as significant independent predictors of Step 1 performance. We also replicated the associations of Step 1 scores with MCAT scores, preclinical grades, and test anxiety, thereby increasing our confidence in the validity of this study.

The use of boards-style questions and Anki flashcards predicted performance on Step 1 in our multivariate model, while Firecracker use did not. The magnitude of improvement in score per question differed between boards-style questions and Anki flashcards. These results are consistent with the concept that retrieval practice methods vary in their benefit to the learner based on question type, corrective feedback, and content [26–28]. For example, most boards-style question banks feature ‘context-rich multiple choice questions’ that present a clinical case and require multiple steps of knowledge retrieval and application, a format that may enhance learning per question compared with single-step, ‘factoid’ questions [28]. Factors that could contribute to the difference in effect between Anki and Firecracker include frequency of repetitions, information complexity on each flashcard, similarity of flashcard content with material on Step 1, and additional learning gained in the process of creating flashcards.

Test anxiety was strongly negatively correlated with Step 1 performance, MCAT score, and preclinical course performance. In the regression model, test anxiety was also the only predictor that demonstrated a statistically significant negative effect on Step 1 score when controlling for other variables. In comparison, depressive symptoms were not independent predictors of Step 1 performance. These results are consistent with other recent studies [23, 29]. Therefore, educators interested in improving test performance may want to focus on students with significant test anxiety. Unfortunately, while certain interventions can reduce test-related anxiety [30], there is little evidence regarding their impact on test performance [23].

Our study has several limitations. First, the sample of students at a single institution may have unique characteristics that limit the generalizability of the results. This study should be replicated in other cohorts. Second, we found evidence for a small degree of response bias based on Step 1 scores, so our results may not fully generalize to lower-scoring students. However, the majority of students participated and our response rate compared favourably with other medical education studies [31]. Third, the data were self-reported. As the survey was anonymous, there was no incentive to falsify responses, but unconscious recall bias remains possible. We attempted to improve precision in recall of number of questions or flashcards completed by providing respondents with the number of items available in each commercial product. Given that our data cannot be independently confirmed, our results must be considered exploratory and interpreted with caution. Fourth, unmeasured forms of Step 1 preparation may confound our analysis. For example, students who complete more practice questions or flashcards may simply study more in general, though the lack of a benefit with Firecracker compared with Anki suggests against this confounder. Similarly, students with attributes associated with improved test performance, such as concentration or self-directedness [12], may be more likely to use more practice questions or Anki flashcards and also be more likely to score higher on Step 1 via other mechanisms. More direct causal inferences on the effects of these self-testing resources require a prospective randomized trial. Fifth, in order to maximize survey completion rates, we used abbreviated versions of questionnaires on psychological well-being and test anxiety. While the reliability coefficients were acceptable in the current study, future works may benefit from using longer instruments that are broadly validated. Despite these limitations, our data are both internally consistent and are supported by a broad body of literature. Therefore, we feel that our results make an important contribution to the discussion of how students can best prepare for high-stakes examinations.

## Conclusions

Our study demonstrates that medical students engage extensively in self-initiated retrieval practice, with or without spaced repetition, in preparation for USMLE Step 1. These practices are associated with superior performance, though the level of benefit varies by question type. Retrieval practice methods should be considered for further study and support by medical schools. Formal prospective evaluation of retrieval practice schedules and approaches in more diverse cohorts should be pursued in order to clarify and confirm effective applications of the technique.

## Essentials

- Retrieval practice, the act of recalling information through questions or testing, strengthens memory.
- Study resources that harness retrieval practice include banks of context-rich multiple choice questions as well as free recall flashcards delivered via spaced repetition software.
- Contemporary preclinical medical students at one institution engaged extensively in self-initiated retrieval practice, sometimes with spaced repetition.
- Students who used more practice questions and flashcards on certain spaced repetition platforms performed better on a cumulative medical licensing examination.
