# Erratum to: Student-directed retrieval practice is a predictor of medical licensing examination performance

**DOI:** 10.1007/s40037-016-0312-2

**Published:** 2016-11-18

**Authors:** Francis Deng, Jeffrey A. Gluckstein, Douglas P. Larsen

**Affiliations:** 1 Brigham and Women’s Hospital, Harvard Medical School, 02115 75 Francis St, Boston, MA USA; 2 Department of Neurology, Washington University School of Medicine, Saint Louis, MO USA

**Erratum to:**

**Perspect Med Educ (2015) 4:308–313**

**DOI 10.1007/s40037-015-0220-x**

In the version of the article originally published online, there was an error in Table 3 (page 312). The B coefficient for the variable “Anki” should be 5.89 × 10^−4^ rather than 5.89 × 10^−3^. The exponent is correctly reported in the body text (page 311).

